# Younger age of escalation of cardiovascular risk factors in Asian Indian subjects

**DOI:** 10.1186/1471-2261-9-28

**Published:** 2009-07-05

**Authors:** Rajeev Gupta, Anoop Misra, Naval K Vikram, Dimple Kondal, Shaon Sen Gupta, Aachu Agrawal, RM Pandey

**Affiliations:** 1 Department of Medicine, Fortis Escorts Hospital, Jaipur 302017, India; 2 Department of Diabetes and Metabolic Diseases, Fortis Rajan Dhall Hospital, New Delhi 110055, India; 3 Department of Medicine, All India Institute of Medical Sciences, New Delhi 110029, India; 4 Department of Biostatistics, All India Institute of Medical Sciences, New Delhi 110029, India; 5 Department of Home Science, University of Rajasthan, Jaipur 302004, India

## Abstract

**Background:**

Cardiovascular risk factors start early, track through the young age and manifest in middle age in most societies. We conducted epidemiological studies to determine prevalence and age-specific trends in cardiovascular risk factors among adolescent and young urban Asian Indians.

**Methods:**

Population based epidemiological studies to identify cardiovascular risk factors were performed in North India in 1999–2002. We evaluated major risk factors-smoking or tobacco use, obesity, truncal obesity, hypertension, dysglycemia and dyslipidemia using pre-specified definitions in 2051 subjects (male 1009, female 1042) aged 15–39 years of age. Age-stratified analyses were performed and significance of trends determined using regression analyses for numerical variables and Χ^2 ^test for trend for categorical variables. Logistic regression was used to identify univariate and multivariate odds ratios (OR) for correlation of age and risk factors.

**Results:**

In males and females respectively, smoking or tobacco use was observed in 200 (11.8%) and 18 (1.4%), overweight or obesity (body mass index, BMI ≥ 25 kg/m^2^) in 12.4% and 14.3%, high waist-hip ratio, WHR (males > 0.9, females > 0.8) in 15% and 32.3%, hypertension in 5.6% and 3.1%, high LDL cholesterol (≥ 130 mg/dl) in 9.4% and 8.9%, low HDL cholesterol (<40 mg/dl males, <50 mg/dl females) in 16.2% and 49.7%, hypertriglyceridemia (≥ 150 mg/dl) in 9.7% and 6%, diabetes in 1.0% and 0.4% and the metabolic syndrome in 3.4% and 3.6%. Significantly increasing trends with age for indices of obesity (BMI, waist, WHR), glycemia (fasting glucose, metabolic syndrome) and lipids (cholesterol, LDL cholesterol, HDL cholesterol) were observed (p for trend < 0.01). At age 15–19 years the prevalence (%) of risk factors in males and females, respectively, was overweight/obesity in 7.6, 8.8; high WHR 4.9, 14.4; hypertension 2.3, 0.3; high LDL cholesterol 2.4, 3.2; high triglycerides 3.0, 3.2; low HDL cholesterol 8.0, 45.3; high total:HDL ratio 3.7, 4.7, diabetes 0.0 and metabolic syndrome in 0.0, 0.2 percent. At age groups 20–29 years in males and females, ORs were, for smoking 5.3, 1.0; obesity 1.6, 0.8; truncal obesity 4.5, 3.1; hypertension 2.6, 4.8; high LDL cholesterol 6.4, 1.8; high triglycerides 3.7, 0.9; low HDL cholesterol 2.4, 0.8; high total:HDL cholesterol 1.6, 1.0; diabetes 4.0, 1.0; and metabolic syndrome 37.7, 5.7 (p < 0.05 for some). At age 30–39, ORs were- smoking 16.0, 6.3; overweight 7.1, 11.3; truncal obesity 21.1, 17.2; hypertension 13.0, 64.0; high LDL cholesterol 27.4, 19.5; high triglycerides 24.2, 10.0; low HDL cholesterol 15.8, 14.1; high total:HDL cholesterol 37.9, 6.10; diabetes 50.7, 17.4; and metabolic syndrome 168.5, 146.2 (p < 0.01 for all parameters). Multivariate adjustment for BMI, waist size and WHR in men and women aged 30–39 years resulted in attenuation of ORs for hypertension and dyslipidemias.

**Conclusion:**

Low prevalence of multiple cardiovascular risk factors (smoking, hypertension, dyslipidemias, diabetes and metabolic syndrome) in adolescents and rapid escalation of these risk factors by age of 30–39 years is noted in urban Asian Indians. Interventions should focus on these individuals.

## Background

Cardiovascular diseases in developing countries are characterized by early age of onset and greater mortality [1]. Worldwide data reveal that while more than 70% of deaths in high income countries occur after the age of 70 years, in low and middle income countries more than 70 percent deaths occur below this age [2,3]. Coronary heart disease occurs at least ten years earlier in South Asians as compared to other ethnic groups and the average age of stroke is much lower than in the western countries. The INTERHEART Study [4] reported that the mean age of myocardial infarction in South Asians was 52 years as compared to 60–65 years in European and North American cohorts. This study also reported that usual cardiovascular risk factors important in older subjects were equally important in the young South Asians [4].

Cardiovascular risk factors have been studied in the younger populations-children, adolescents, and youth in different parts of the world [5]. These studies have been mainly confined to high income countries of North America, Europe and Australasia. Examples from USA include the Bogalusa Heart Study in Caucasians and Black Americans [6], National Health and Nutrition Examination Surveys (NHANES) [7], Muscatine Study [8], Coronary Artery Risk Development in Young Adults (CARDIA) study [9], Atherosclerosis Risk in Young Adults (ARYA) study [10], Atherosclerosis Risk Factors in Male Youngsters (ARMY) study [11], Pathobiological Determinants of Atherosclerosis in Young (PDAY) study [12], Minneapolis Children's Blood Pressure Study [13], and in Europe the Cardiovascular Risk in Young Finns study [14], North Ireland Young Heart Project [15] and many British studies [16]. All these studies have reported that the atherosclerosis factors start early in childhood and youth and the risk factors tend to track and magnify with age. The mean age at which the risk factors establish varies from 40–50 years [17-19].

Young subjects have not been well studied from low income countries. In India some studies have reported a moderate to high prevalence of multiple atherosclerosis risk factors in the adolescents and the young [20,21]. Studies from metropolitan cities in non-governmental schools report a high prevalence of obesity [22]. In low-income schools in Indian urban and rural areas there is a low prevalence of obesity but high prevalence of smoking and tobacco use [23]. No studies that systematically evaluate these risk factors with age have been performed. Such studies are essential to develop cost-effective screening and intervention programs. We performed population based cross-sectional studies in two North Indian cities to study cardiovascular risk factors in the young subjects 15 to 39 years of age. The aims of the study were to determine prevalence of multiple cardiovascular risk factors in the adolescent and young adults and to study the age-group where risk factor determinants such as obesity and central obesity emerge and association of these with prevalence of high blood pressure, lipid abnormalities, diabetes, and metabolic syndrome.

## Methods

To determine prevalence of various cardiovascular risk factors in adolescents and young adults, we performed population-based epidemiological studies in two north Indian cities, Jaipur and Delhi. The studies were approved by respective institutional ethics committees. These epidemiological population based studies were conducted between 1999 and 2002 in these cities by two investigators. One study was conducted in New Delhi that included subjects aged 14–25 years drawn from an approximate total population of 15,000 subjects from schools and colleges of southwest Delhi using multistage cluster sampling based on World Health Organization (WHO) Expanded Program of Immunization Sampling Plan as described elsewhere [24]. The data of subjects in age-group 15–25 years (n = 1680, 865 males, 815 females) were included for present analysis. The second study was conducted in the city of Jaipur in northwestern India using previously published methodology [25]. Total population of Jaipur in more than 23 million and the study was performed in six localities of Jaipur with population of 130,000 and 1123 subjects of the target 1800 (response 62.4%) were evaluated. In the present analysis, the data of subjects belonging to the age group 20–39 years (n = 371, 194 males, 177 females) were included. Informed consent was obtained from parents and school authorities in children ≤ 18 years of age and from all the adult subjects according to the ethics committee guidelines. History of previously diagnosed hypertension, diabetes or other diseases and smoking status or tobacco use in other forms was obtained.

Height, weight, waist and hip circumferences measurements were recorded using previously reported methodology at each centre by a single centrally trained observer [24]. Blood pressure (BP) was recorded using a standard mercury sphygmomanometer with the subject seated and rested for five minutes. At least two readings at 5 minute interval were recorded and in case of an abnormal reading, another reading was obtained after 30 minutes. The data of anthropometric parameters (height, weight, waist size, and hip size) and blood pressure measurements were available for all subjects. Central training for technicians was performed to achieve uniformity in measurements and data collection. Height and weight were measured with calibrated instruments, waist was measured using a non-stretch steel tape measure at the mid-point of lower ribs and iliac crest in mid expiration in standing position and hip circumference was measured at inter-trochanteric level as reported earlier [24]. Fasting blood sample was obtained after an overnight fast for estimation of glucose, total cholesterol, high density lipoprotein (HDL) cholesterol and triglycerides using previously reported methodologies [24,25]. Similar equipment and diagnostic reagents were used at both the centres to achieve standardization in biochemical measurements. Low density lipoprotein (LDL) cholesterol was calculated using Freidewald's formula.

### Definitions

Cardiovascular risk factors studied were: smoking, obesity, high waist circumference (WC), hypertension, glucose intolerance, dyslipidemia (high LDL cholesterol, hypertriglyceridemia, low HDL cholesterol or high total-HDL cholesterol ratio). Overweight or obesity was defined as body mass index (BMI) ≥ 25 kg/m^2^. Among the measures of abdominal obesity, high WC was defined as >90 cm in males and >80 cm in females and high waist:hip ratio (WHR) was defined as >0.9 in males and >0.8 in females. Hypertension was defined as a persistent elevation of blood pressure ≥140/≥90 mm Hg or use of any anti-hypertensive medication. Impaired fasting glucose (IFG) was defined as fasting blood glucose value of ≥ 100 mg/dl and ≤125 mg/dl, and diabetes was defined as fasting blood glucose value ≥126 mg/dl or on any treatment. Dyslipidemias were defined according to the USA National Cholesterol Education Program (NCEP) criteria (high LDL cholesterol as ≥ 130 mg%, hypertyriglyceridemia as ≥ 150 mg/dl and low HDL cholesterol as <40 mg/dl in males and <50 mg/dl in females) [26]. High total cholesterol: HDL ratio was defined as >4.5. Metabolic syndrome was defined according to the NCEP definition with modified WC cut off values (males > 90 cm, females > 80 cm) [27].

### Statistical analysis

For the purpose of analysis both the data sets were combined into one. Approximate normality was assessed for anthropometric and biochemical parameters. SPSS 10.0 and STATA 9.0 statistical softwares were used for data analyses. Quantitative characteristics were summarized by arithmetic mean and standard deviation. The differences in the anthropometric and biochemical parameters were compared using Z-test. Age-specific mean values of various anthropometric and biochemical characteristics were compared using one way ANOVA followed by post-hoc Bonferroni test. Anthropometric and biochemical characteristics were further categorized according to the cut off values as mentioned in the definition section. Categorical variables were summarized by percentages. Numerical trends in various cardiovascular risk factors across different age groups were assessed using regression analyses using CURVEFIT command of SPSS (version 10.0). Regression coefficients (B), standard deviations, standardized regression coefficients (beta), and p values were estimated. *Χ*^2 ^test for trend for performed for ordinal variables using the SPSS and are reported with p values. To determine the age associated escalation of risk factors odds ratios were calculated using logistic regression analysis. Univariate odds ratios (OR) and 95% confidence intervals (CI) were calculated with age-group 15–19 as baseline and compared with age-group 20–29 and 30–39 years. To determine significance of different measures of obesity (BMI, WC, WHR) multivariate logistic regression analyses were performed after adjustment for these factors. P value < 0.05 was considered statistically significant.

## Results

Data of 2051 subjects aged 15–39 years (males 1009, females 1042) were evaluated in the present study. Subjects in different age groups were: 15–19 years 1292 (63.0%, 701 males and 591 females), 20–29 years 721 (20.5%, 174 males and 281 females), and 30–39 years 270 (6.0%, 59 males and 65 females). The prevalence of smoking was 6.6%, being higher in males (11.8%) than in females (1.4%). Various physical and biochemical characteristics of the study population are presented in Table 1. Mean BMI was similar in both males and females while WC, WHR and systolic and diastolic blood pressures were significantly higher in males as compared to females (p < 0.01). Mean fasting blood glucose was higher in males as compared to females (p < 0.01). The mean levels of total cholesterol, HDL cholesterol and LDL cholesterol were significantly higher in females (p < 0.01) whereas triglycerides levels were higher in males (p < 0.05). Obesity (BMI ≥ 25 kg/m^2^) was present in 13.4% (males 12.4%, females 14.3%), high WC in 13.3% (males 11.9%, females 14.7%), high WHR in 21.2% (males 13.7%, females 28.4%) and hypertension in 4.3% (males 5.6%, females 3.1%) subjects. Diabetes (known or diagnosed) was present in very small proportion (0.7%) of subjects, but impaired fasting glucose (100–125 mg/dl) was present in 10.8% (13.4% males, 8.2% females). Among the dyslipidemias, hypercholesterolemia was present in 7.9% (8% males, 7.8% females), high LDL cholesterol (≥ 130 mg/dl) in 9% (9.2% males, 8.7% females), hypertriglyceridemia in 7.9% (9.7% males, 6% females), low HDL cholesterol in 33.2% (16.2% males, 49.7% females) and high total:HDL cholesterol ratio in 13.5% (14.3% males, 12.7% females) subjects (Table 1).

**Table 1:** Physical and Biochemical Characteristics in the Study Subjects

**Variables**	**Males (n = 1009)**	**Females (n = 1042)**	**Overall (n = 2051)**
Weight (kg)	57.8 ± 13.2	50.4 ± 10.1*	54.0 ± 12.3
Body mass index (kg/m^2^)	20.6 ± 4.1	20.7 ± 3.9	20.7 ± 4.0
Obesity (BMI kg/m^2^)			
23.0–24.9	108 (10.7)	94 (9.0)	202 (9.8)
25.0–29.9	93 (9.2)	114 (10.9)	207 (10.1)
≥ 30	32 (3.2)	35 (3.4)	67 (3.3)
Waist (cm)	74.4 ± 12.1	70.0 ± 10.4*	72.2 ± 11.5
Waist size (cm)			
70–79	284 (28.1)	281 (27.0)	565 (27.5)
80–89	153 (15.2)	102 (9.8)	255 (12.4)
90–99	79 (7.8)	45 (4.3)	124 (6.0)
≥ 100	45 (4.5)	16 (1.5)	61 (3.0)
Waist:hip ratio	0.83 ± 0.06	0.77 ± 0.07*	0.80 ± 0.07
Waist:hip ratio			
0.70–0.799	289 (28.6)	581 (55.6)	870 (42.4)
0.80–0.899	567 (56.2)	295 (28.3)	862 (42.0)
0.90–0.999	130 (12.9)	39 (3.7)	169 (8.2)
≥ 1.00	21 (2.1)	3 (0.3)	24 (1.2)
			
Systolic BP (mm Hg)	115.9 ± 10.9	111.3 ± 10.7*	113.6 ± 11.0
Diastolic BP (mm Hg)	74.9 ± 8.2	73.2 ± 7.7*	74.0 ± 8.0
Hypertension (≥ 140/90 or known)	57 (5.6)	32 (3.1)*	89 (4.3)
Fasting glucose (mg/dl)	89.9 ± 13.9	88.3 ± 10.9*	89.1 ± 12.5
Glucose fasting (mg/dl)			
90–99	370 (36.7)	379 (36.4)	49 (36.5)
100–109	119 (11.8)	81 (7.8)	200 (9.8)
110–125	16 (1.6)	4 (0.4)	20 (1.0)
Diabetes (>125 mg/dl or known)	10 (1.0)	4 (0.4)	14 (0.7)
Cholesterol (mg/dl)	153.5 ± 37.6	162.7 ± 29.7*	158.7 ± 49.7
LDL cholesterol (mg/dl)	87.1 ± 34.7	93.2 ± 30.3*	90.2 ± 32.7
High LDL cholesterol (mg/dl)			
100–129	214 (21.2)	297 (28.5)	511 (24.9)
130–159	56 (5.6)	62 (6.0)	118 (5.7)
≥ 160	38 (3.8)	30 (2.9)	68 (3.3)
Triglycerides (mg/dl)	102.0 ± 58.2	97.4 ± 40.3**	99.7 ± 50.0
High triglycerides (mg/dl)			
150–199	58 (5.7)	40 (3.8)	98 (4.8)
200–399	35 (3.5)	22 (2.1)	57 (2.8)
≥ 400	5 (0.5)	1 (0.1)	6 (0.3)
HDL cholesterol (mg/dl)	46.1 ± 7.85	49.9 ± 9.79*	48.05 ± 9.09
Low HDL cholesterol (males < 40 mg/dl and females < 50 mg/dl)	163 (16.2)	518 (49.7)*	681 (33.2)
Total: HDL cholesterol	3.48 ± 1.37	3.43 ± 1.18	3.45 ± 1.27
High total: HDL cholesterolcratio ≥ 4.5	144 (14.3)	132 (12.7)	276 (13.5)

*: p < 0.01; **: p < 0.05. Values are expressed as Mean ± SD or numbers with percent in parentheses.

**Figure 1:**
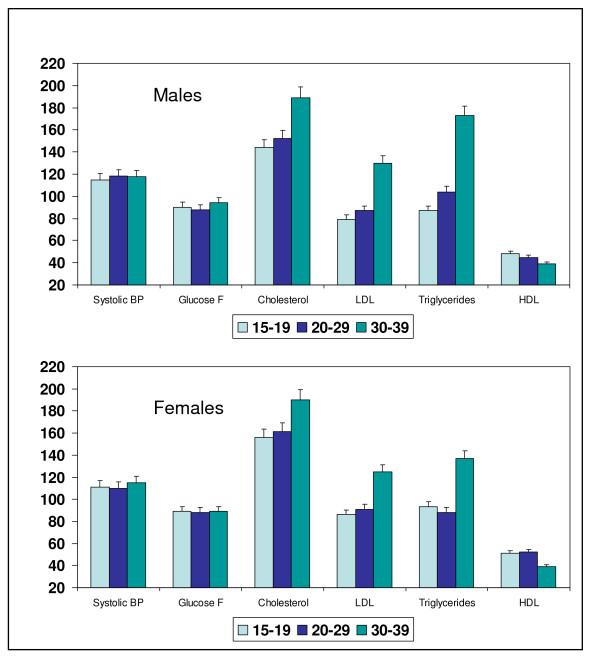
**Age-related escalation in mean levels of systolic BP, fasting glucose, total cholesterol, LDL cholesterol and triglycerides and decline in HDL cholesterol levels in males and females (p for trend < 0.01)**. Values of systolic BP are in mm Hg and the others in mg/dl. Bars and whiskers indicate mean and SEM values respectively.

Age-specific mean values of various clinical, anthropometric and biochemical characteristics are shown in Table 2. Both in males and females significantly increasing trend in BMI, WC, WHR, systolic BP, cholesterol, LDL cholesterol, triglycerides and a decreasing trend in HDL cholesterol levels is observed (regression analyses, p < 0.001). In both males and females there is a steep increase in mean BMI, WC, and WHR as these men move from age-groups 20–29 to 30–39 years. Similar increasing trends are observed in mean levels of total- and LDL cholesterol and triglycerides with declining trends in HDL cholesterol levels (Figure 1, p < 0.001). Age-related trends in the prevalence of various cardiovascular risk factors are presented in Table 3. In both men and women the prevalence of obesity is low in age groups 15–20 and 20–29 years with a steep increase in age-group 30–39 years (men 36.8%, women 52.1%) (*Χ*^2 ^test, p for trend < 0.001). Similar changes are observed in the prevalence of high WC and high WHR (p for trend < 0.001). A significantly increasing trend in prevalence of hypertension, impaired fasting glucose, high LDL cholesterol, hypertriglyceridemia, high total:HDL cholesterol ratio, low HDL cholesterol, diabetes and the metabolic syndrome is also observed.

**Table 2:** Cardiovascular Risk Factors at Different Age-Groups (Mean ± SD) and Trends

**Variables**	**Males**				**Females**			
	15–19 years (n = 699)	20–29 years (n = 175)	30–39 years (n = 133)	Regression coefficient, standardized beta, p-value	15–19 years (n = 591)	20–29 years (n = 311)	30–39 years (n = 140)	Regression coefficient, standardized beta, p-value
Body mass index (kg/m^2^)	19.7 ± 3.3	21.5 ± 4.0	24.1 ± 5.6	2.11 ± 0.17,0.37, <0.001	20.0 ± 3.2	20.2 ± 3.3	24.7 ± 5.1	1.80 ± 0.16, 0.332, <0.001
Waist circumference (cm)	70.6 ± 9.2	77.7 ± 10.8	89.6 ± 14.0	9.04 ± 0.45, 0.53, <0.001	66.8 ± 7.7	69.9 ± 9.1	83.8 ± 11.8	7.12 ± 0.39, 0.491, <0.001
Waist-hip ratio	0.81 ± 0.05	0.85 ± 0.09	0.91 ± 0.07	0.047 ± 0.03, 0.48, <0.001	0.75 ± 0.06	0.78 ± 0.06	0.84 ± 0.07	0.04 ± 0.01, 0.457, <0.001
Systolic blood pressure (mm Hg)	115.0 ± 9.6	118.2 ± 10.8	117.3 ± 15.9	1.56 ± 0.48, 0.10, 0.001	111.0 ± 9.0	110.4 ± 9.5	114.5 ± 17.0	1.16 ± 0.46, 0.078, 0.011
Diastolic blood pressure (mm Hg)	74.4 ± 7.2	75.6 ± 8.2	76.5 ± 11.9	1.07 ± 0.36, 0.09, 0.003	72.9 ± 6.9	72.8 7.2	75.0 ± 11.2	0.74 ± 0.33, 0.069, 0.025
Glucose fasting (mg/dl)	90.0 ± 9.2	86.7 ± 2.5	94.1 ± 28.2	1.02 ± 0.61, 0.05, 0.096	88.7 ± 8.3	87.7 ± 8.3	88.4 ± 21.0	-0.36 ± 0.47, -0.024, 0.441
Total cholesterol (mg/dl)	144.4 ± 26.0	151.9 ± 38.9	203.9 ± 47.6	25.51 ± 1.5, 0.48, <0.001	156.4 ± 23.0	160.9 25.7	193.0 ± 41.0	14.81 ± 1.19, 0.361, <0.001
LDL cholesterol (mg/dl)	79.0 ± 25.9	86.8 ± 2.5	130.0 ± 40.9	22.11 ± 1.4, 0.45, <0.001	86.5 ± 24.3	90.9 ± 26.7	126.6 ± 38.0	16.14 ± 1.21, 0.382, <0.001
Triglycerides (mg/dl)	87.5 ± 30.1	104.3 ± 39.9	173.5 ± 111.5	37.99 ± 2.3, 0.47, <0.001	92.7 ± 28.0	88.2 ± 29.5	137.3 ± 70.6	15.54 ± 1.67, 0.277, <0.001
HDL cholesterol (mg/dl)	47.7 ± 6.8	45.1 ± 7.9	39.2 ± 9.0	-3.94 ± 0.32, -0.36, <0.001	51.1 ± 8.6	52.6 ± 6.2	39.0 ± 8.5	-4.14 ± 0.40, -0.303, <0.001
Total: HDL cholesterol ratio	3.1 ± 0.70	3.5 ± 1.4	5.5 ± 2.1	1.06 ± 0.05, 0.55, <0.001	3.2 ± 0.73	3.2 ± 0.85	5.2 ± 1.72	0.77 ± 0.045, 0.468, <0.001

**Table 3:** Prevalence of Various Cardiovascular Risk Factors at Different Age-Groups and Trends

**Variables**	**Males**				**Females**			
	15–19 years (n = 699)	20–29 years (n = 175)	30–39 years (n = 133)	*X*^2 ^for trend (p-value)	15–19 years (n = 591)	20–29 years (n = 311)	30–39 years (n = 140)	*X*^2 ^for trend (p-value)
Body mass index ≥ 25 Kg/m2	53 (7.6)	20 (11.5)	49 (36.8)	89.85 (<0.001)	52 (8.8)	23 (7.4)	73 (52.1)	191.09 (<0.001)
Body mass index ≥ 30 Kg/m2	7 (1.0)	7 (4.0)	17 (12.8)	52.55 (<0.001)	5 (0.8)	7 (2.3)	22 (15.7)	80.62 (<0.001)
Waist circumference > 90 cm/80 cm	32 (4.6)	20 (11.4)	67 (50.4)	224.94 (<0.001)	36 (6.1)	31 (10.0)	86 (61.4)	284.55 (<0.001)
Waist hip ratio > 0.9/0.8	34 (4.9)	33 (18.9)	69 (51.9)	216.64 (<0.001)	85 (14.4)	107 (34.4)	104 (74.3)	207.56 (<0.001)
Hypertension	16 (2.3)	10 (5.7)	31 (23.3)	92.45 (<0.001)	2 (0.3)	5 (1.6)	25 (17.9)	119.89 (<0.001)
Impaired fasting glucose (100–125 mg/dl)	99 (14.2)	10 (5.7)	26 (19.5)	13.59 (0.001)	49 (8.3)	20 (6.4)	16 (11.4)	3.25 (0.197)
Diabetes mellitus (≥ 126 mg/dl)	-	1 (0.6)	9 (6.8)	52.43 (<0.001)	-	-	4 (2.9)	25.87 (<0.001)
LDL cholesterol ≥ 130 mg/dl	17 (2.4)	24 (13.7)	54 (40.6)	195.08 (<0.001)	19 (3.2)	18 (5.8)	55 (39.3)	188.05 (<0.001)
Triglycerides ≥ 150 mg/dl	21 (3.0)	18 (10.3)	57 (42.9)	205.92 (<0.001)	19 (3.2)	9 (2.9)	35 (25.0)	102.32 (<0.001)
HDL cholesterol <40 mg/dl	56 (8.0)	30 (17.1)	77 (57.9)	205.09 (<0.001)	268 (45.3)	120 (38.6)	129 (92.1)	120.73 (<0.001)
Total: HDL cholesterol ratio ≥ 4.5	26 (3.7)	30 (17.1)	79 (59.4)	300.95 (<0.001)	28 (4.7)	22 (7.1)	82 (58.6)	309.04 (<0.001)
Metabolic syndrome	-	9 (5.1)	26 (19.5)	129.04 (<0.001)	1 (0.2)	3 (1.0)	28 (20.0)	156.14 (<0.001)
Numbers in parentheses are percent.								

**Figure 2:**
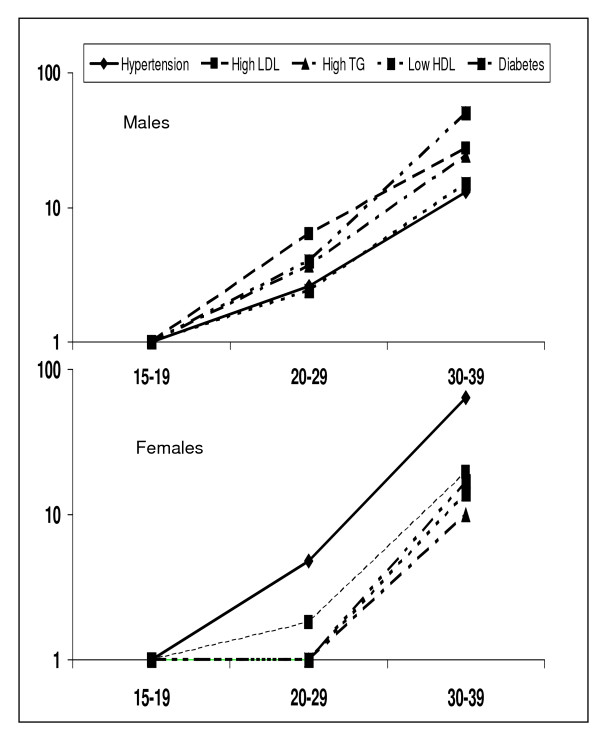
**Trends of increase in prevalence of hypertension, high LDL cholesterol, high triglyceride (TG) levels, low HDL cholesterol and diabetes with age in males and females**. Relative prevalence of risk factors at age-groups 15–19 (odds ratio = 1), 20–29 and 30–39 is shown. The increase is slow from age 15–19 to 20–29 years with an exponential increase at age-group 30–39 years (p < 0.001).

Regression analyses reveal that age is an important determinant of blood pressure as well as multiple biochemical factors (Table 4). BMI, WC as well as WHR are significantly positively related with systolic BP, total cholesterol, LDL cholesterol, and triglycerides and negatively with HDL cholesterol. Age-adjustment reveals that while BMI is a more important determinant of systolic BP, waist circumference and waist-hip ratio are more important for biochemical abnormalities. All the associations loose significance when adjusted for age, BMI and measures of truncal obesity (Table 4). Trends of risk factors change with age using univariate and multivariate odds ratios are shown in Table 5. At age 15–19 years the prevalence (%) of risk factors in males and females, respectively, was overweight/obesity in 7.6, 8.8; high WHR 4.9, 14.4; hypertension 2.3, 0.3; high LDL cholesterol 2.4, 3.2; high triglycerides 3.0, 3.2; low HDL cholesterol 8.0, 45.3; high total:HDL ratio 3.7, 4.7, diabetes 0.0 and metabolic syndrome in 0.0, 0.2 percent. In males and females, respectively, as compared to age group 15–19 years, ORs for risk factors at age groups 20–29 years are for smoking 5.3, 1.0; obesity 1.6, 0.8; truncal obesity 4.5, 3.1; hypertension 2.6, 4.8; high LDL cholesterol 6.4, 1.8; high triglycerides 3.7, 0.9; low HDL cholesterol 2.4, 0.8; high total:HDL cholesterol 1.6, 1.0; diabetes 4.0, 1.0; and the metabolic syndrome 37.7, 5.7 (Table 5). The escalation is highly significant at age 30–39 with ORs for smoking 16.0, 6.3; overweight 7.1, 11.3; truncal obesity 21.1, 17.2; hypertension 13.0, 64.0; high LDL cholesterol 27.4, 19.5; high triglycerides 24.2, 10.0; low HDL cholesterol 15.8, 14.1; high total:HDL cholesterol 37.9, 6.10; diabetes 50.7, 17.4; and metabolic syndrome 168.5, 146.2 (Figure 2, p < 0.01 for all parameters). Multivariate adjustment for BMI, waist size and WHR in men and women aged 30–39 years results in attenuation in ORs for hypertension and various lipid parameters but remain significant.

**Table 4:** Standardized Regression Coefficients for Association of Cardiovascular Risk Factors with Age and Different Measures of Obesity.

	**Age**	**Body Mass Index**			**Waist Circumference**			**Waist Hip Ratio**		
		Univariate	Age-Adjusted	Multivariate adjusted for age, waist, WHR	Univariate	Age-Adjusted	Multivariate adjusted for age, body mass index, WHR	Univariate	Age-Adjusted	Multivariate adjusted for age, body mass index, waist

Age	-	0.394***	-	-	0.536***	-	-	0.417***	-	-

Systolic BP	0.092***	0.311***	0.375***	0.145***	0.335***	0.402***	0.207***	0.261***	0.269***	0.099**

Cholesterol	0.468***	0.283***	0.117***	-0.012	0.340***	0.125***	0.236***	0.192***	-0.004	-0.142***

LDL cholesterol	0.459***	0.276***	0.113***	-0.018	0.341***	0.133***	0.227***	0.208***	0.020	-0.111***

HDL cholesterol	-0.337***	-0.195***	-0.073**	0.094*	-0.302***	-0.171***	-0.202***	-0.275***	-0.163***	-0.069*

Triglycerides	0.414***	0.246***	0.098***	-0.028	0.325***	0.144***	0.183**	0.240***	0.081*	-0.021

Glucose Fasting	0.038	0.026	0.013	-0.073	0.056*	0.049	0.127*	0.044*	0.034	-0.020

BP blood pressure; WHR waist hip ratio; * p < 0.01; ** p < 0.001; ** p < 0.001;

**Table 5:** Relative prevalence of various risk factors with increasing age in males and females presented as univariate and adjusted odds ratios and 95% confidence intervals

**Variables**	**Age group**	**Univariate odds ratios**	**Adjusted for body mass index**	**Adjusted for waist circumference**	**Adjusted for waist hip ratio**
**Males**					

Smoking	15–19	1.0	1.0	1.0	1.0

	20–29	5.3 (3.6–7.0)	--	--	--

	30–39	16.0 (10.5–21.5)	--	--	--

Body mass index ≥ 25 kg/m2	15–19	1.0	--	1.0	1.0

	20–29	1.6 (0.9–2.7)	--	--	--

	30–39	7.1 (4.5–11.2)	--	--	--

Waist:hip ratio > 0.9	15–19	1.0	1.0	--	--

	20–29	4.5 (2.7–7.6)	--	--	--

	30–39	21.1 (12.9–34.2)	--	--	--

Hypertension	15–19	1.0	1.0	1.0	1.0

	20–29	2.6 (1.2–5.8)	1.7 (0.7–3.9)	1.1 (0.4–2.8)	1.3 (0.5–3.2)

	30–39	13.0 (6.9–24.6)	7.4 (3.7–14.8)	3.4 (1.5–7.5)	4.3 (1.9–9.5)

LDL cholesterol ≥ 130 mg/dl	15–19	1.0	1.0	1.0	1.0

	20–29	6.4 (3.3–12.2)	5.9 (3.0–11.5)	4.7 (2.4–9.4)	5.4 (2.7–10.9)

	30–39	27.4 (15.2–49.6)	22.2 (11.7–42.0)	16.7 (8.2–34.1)	20.1 (9.8–41.3)

Triglycerides ≥ 150 mg/dl	15–19	1.0	1.0	1.0	1.0

	20–29	3.7 (1.9–7.1)	2.7 (1.4–5.4)	2.4 (1.2–4.8)	2.6 (1.3–5.1)

	30–39	24.2 (13.9–42.1)	19.9 (10.9–36.1)	15.8 (8.2–30.8)	16.8 (8.6–32.6)

HDL cholesterol < 40 mg/dl	15–19	1.0	1.0	1.0	1.0

	20–29	2.4 (1.5–3.8)	2.4 (1.5–3.9)	1.9 (1.2–3.3)	1.7 (1.0–2.9)

	30–39	15.8 (10.2–24.5)	15.1 (9.3–24.7)	12.9 (7.4–22.6)	10.9 (6.4–18.9)

Total: HDL cholesterol > 4.5	15–19	1.0	1.0	1.0	1.0

	20–29	1.6 (1.4–1.9)	1.5 (1.3–1.8)	1.4 (1.1–1.7)	1.4 (1.2–1.7)

	30–39	37.9 (22.5–63.9)	32.7 (18.6–57.5)	27.9 (14.8–52.4)	26.8 (14.3–50.3)

Diabetes	15–19	1.0	1.0	1.0	1.0

	20–29	4.0 (0.3–64.5)	4.2 (0.3–69.9)	2.6 (1.0–72.3)	2.4 (0.9–67.0)

	30–39	50.7 (6.4–403.4)	27.8 (3.2–242.7)	26.2 (1.9–365.8)	19.9 (1.2–336.1)

Metabolic syndrome	15–19	1.0	1.0	1.0	1.0

	20–29	37.7 (4.8–298.2)	20.7 (2.5–171.7)	14.3 (1.6–128.4)	17.3 (2.1–148.5)

	30–39	168.5 (22.8–1247.1)	95.6 (12.3–741.5)	62.9 (6.4–621.3)	59.1 (6.2–568.1)

**Females**					

Smoking	15–19	1.0	1.0	1.0	1.0

	20–29	1.0	--	--	--

	30–39	6.3 (5.5–7.1)	--	--	--

Body mass index ≥ 25 kg/m2	15–19	1.0	1.0	1.0	1.0

	20–29	0.8 (0.5–1.4)	--	--	--

	30–39	11.3 (7.3–11.5)	--	--	--

Waist:hip ratio > 0.8	15–19	1.0	1.0	1.0	1.0

	20–29	3.1 (2.2–4.3)	--	--	--

	30–39	17.2 (11.0–26.8)	--	--	--

Hypertension	15–19	1.0	1.0	1.0	1.0

	20–29	4.8 (0.9–24.9)	4.5 (0.9–23.7)	3.1 (0.6–16.6)	3.2 (0.6–16.7)

	30–39	64.0 (14.9–73.9)	18.2 (4.1–82.9)	10.3 (2.0–52.0)	39.1 (7.9–192.7

LDL cholesterol ≥ 130 mg/dl	15–19	1.0	1.0	1.0	1.0

	20–29	1.8 (0.9–3.6)	1.8 (0.9–3.5)	1.6 (0.8–3.2)	1.9 (0.9–3.7)

	30–39	19.5 (11.0–34.4)	18.2 (9.5–34.8)	17.2 (8.1–36.7)	19.5 (9.7–39.2)

Triglycerides ≥ 150 mg/dl	15–19	1.0	1.0	1.0	1.0

	20–29	0.9 (0.4–2.0)	0.9 (0.4–2.0)	0.8 (0.3–1.8)	0.8 (0.4–1.9)

	30–39	10.0 (5.5–18.2)	8.5 (4.3–16.6)	7.0 (3.2–15.1)	8.2 (4.0–16.8)

HDL cholesterol < 40 mg/dl	15–19	1.0	1.0	1.0	1.0

	20–29	0.8 (0.6–1.0)	0.8 (0.6–1.0)	0.7 (0.5–0.9)	0.7 (0.6–1.1)

	30–39	14.1 (7.5–26.7)	11.9 (6.1–23.2)	10.6 (5.2–21.8)	15.8 (7.8–32.0)

Total: HDL cholesterol > 4.5	15–19	1.0	1.0	1.0	1.0

	20–29	1.0 (0.9–1.2)	1.0 (0.8–1.2)	0.9 (0.8–1.1)	0.9 (0.8–1.2)

	30–39	6.1 (4.5–8.2)	5.9 (4.3–8.3)	5.3 (3.7–7.6)	5.8 (4.1–8.1)

Diabetes	15–19	1.0	1.0	1.0	1.0

	20–29	1.0	1.0	1.0	1.0

	30–39	17.4 (1.9–156.5)	17.0 (1.3–217.7)	31.6 (0.6–1605.0)	22.0 (1.0–474.5)

Metabolic syndrome	15–19	1.0	1.0	1.0	1.0

	20–29	5.7 (0.6–55.3)	5.2 (0.5–51.1)	2.2 (0.2–23.4)	3.0 (0.3–31.0)

	30–39	146.2 (19.9–1027.9)	75.1 (9.7–583.9)	19.7 (2.5–155.3)	48.8 (6.3–378.9)

## Discussion

This study shows a low prevalence of multiple cardiovascular risk factors (smoking, hypertension, lipid abnormalities and diabetes) among adolescents in an Indian urban population. The prevalence of multiple risk factors increases at age-group 20–29 years with an exponential increase in age group 30–39 years. Increasing risk factors correlate with body mass index, waist circumference and waist-hip ratio. A rapid increase in risk factors in young adulthood identifies the target group for interventions.

Wardle et al reported development of adiposity during adolescence in an ethnically and socioeconomically diverse sample of young people in Britain [28]. It was observed that prevalence of overweight and obesity was high in London school students and there were significant socioeconomic and ethnic inequalities with low socioeconomic status Blacks having the highest prevalence. It was concluded that overweight and obesity was well established by the age of 11 years and prevention efforts need to be targeted early. Obesity among children that tracks into adulthood has also been reported in studies from Europe and USA [5-16]. Gordon-Larsen et al studied five year obesity incidence in the transition period between adolescence and adulthood as part of US National Longitudinal Study of Adolescent Health [29]. It was observed that during the 5-yer transition period the obesity incidence was 12.7% and there was a significant increase in obesity from 10.9% at 18 years of age to 22.1% at young adulthood. Our study on the other hand shows that the transition using similar obesity definitions occurs slightly later than the western populations. Reddy et al studied industrial populations in different parts of India and reported risk factor trends from age groups 20–29 years to ≥60 years [30]. In both men and women there was a steep increase in overweight and central obesity at age-group 30–39 years associated with increase in hypertension and biochemical risk factors. Reasons for the increase in weight beyond 30 years of age could be multiple. Socioeconomic changes that occur at this age in men include financial independence, marriage, transition from an active to sedentary working lifestyle, dietary changes, and psychological changes related to material and social environment [31]. In predominantly home-bound women apart from these changes others include less physical activity with grown-up children, dietary indiscretion and changes material environment [32,33]. We did not study these socioeconomic variables in the present study and hence cannot comment on their importance. Studies in emigrant South Asians to UK have reported similar risk factor trends [34] as in the present study and highlight importance of lifestyle in escalating cardiovascular risks. The INTERHEART study reported that multiple cardiovascular risks occurred at a younger age among South Asians which explained younger age of acute myocardial infarction in these subjects [35]. The present study also shows a younger age of escalation of multiple cardiovascular risk factors and is similar to these studies.

Obesity is a proximate factor for increase in multiple metabolic abnormalities [36]. The present study also shows that development of obesity and central obesity is associated with simultaneous increase in many biological risk factors such as changes in blood pressure levels and glucose and lipid abnormalities. Similar findings have been reported from the Bogalusa Heart Study [6], NHANES [7], and CARDIA study [9] and many other North American and European studies [5]. The present study shows that the increase in risk factors in a North Indian urban population starts at about the age of 30 years and focus of prevention should be at subjects lower than this age.

Several limitations of the present study have to be highlighted. The sample survey for the adults was a population based data collection while for adolescents a school-based survey was performed. This would have excluded the very poor and illiterate children from the study in children in spite of inclusion of illiterates in the adult groups. There is a steep increase in BMI in the age groups ≥30 years in both men and women and inclusion of more poor children with low BMI would have enhanced the already steep change in BMI with increasing age. Secondly, the sample size at different age-groups is not similar and this could have resulted in a bias. However, this is a limitation of population-based study and can be overcome by using purposive age-stratified sampling which was not the design of the present study. Thirdly, this is analysis of cross-sectional data while an ideal study to observe age-related tracking of risk factors involves a prospective design [37]. Prospective studies are expensive and time-consuming and cross-sectional studies such as this are hypothesis generating and most of the time similar conclusions are reached. Fourthly, we used adult BMI criteria for defining overweight and obesity in subjects less than 18 years of age although the criteria are applicable to adults only. However, criteria of >95^th ^percentile for obesity in adolescents are widely debated [29] and for sake of comparability our approach is justified. And finally, multiple ways to analyze secular data are available often involving complex statistics. As the present study was cross-sectional in nature we did not use such analyses.

## Conclusion

India and many developing countries suffer from the double burden of under nutrition and over nutrition [38]. There is an urgent need to improve maternal and childhood nutrition to prevent fetal and childhood stunting and malnutrition related diseases. On the other hand, there is sufficient evidence that problems of under nutrition have been replaced by over nutrition in urban adults in these countries [39]. The crucial issue is age at which screening and intervention strategies should be initiated [40]. The present study shows that dietary and lifestyle changes to prevent adiposity and obesity and consequent epidemic of cardiovascular diseases in India and other such countries can be prevented by targeting men and women at age of about 30 years. A multi-factorial risk screening and intervention approach [41] at the population level could provide immediate benefit.

## Competing interests

The authors declare that they have no competing interests.

## Authors' contributions

RG conceived, designed, overviewed data collection and implemented the study at Jaipur centre, provided critical academic inputs and wrote the first draft of the manuscript and subsequent revisions of the manuscript. AM conceived, designed, overviewed data collection and implemented the study at Delhi centre, provided critical academic inputs and contributed to the first draft of the article and subsequent revisions of the manuscript. NVK also overviewed data collection and implemented the study at Delhi centre, provided critical academic inputs, analysed the data was involved in revisions of the manuscript. DK, SSG and AA were involved in data collection, data entry, statistical analyses and contributed to the manuscript; and RMP helped in study design, overviewed data collection and statistical analyses, and provided critical academic inputs. All authors have read and approved the final manuscript.

## Pre-publication history

The pre-publication history for this paper can be accessed here:


